# Supplementary material for: Genomic diversity and evolution of the Hawaiian Islands endemic Kokia (Malvaceae)
Source: G3 (Bethesda). 2024 Aug 6;14(10):jkae180. doi: 10.1093/g3journal/jkae180 (PMC11457090; doi:10.1093/g3journal/jkae180)
Supplement: jkae180_Supplementary_Data [file jkae180_supplementary_data.zip › Figure_S2_G3-2024-405044.docx]

**Figure S2:** Characteristics of the gene models in the three *Kokia* species *K. cookei* (Kocoo), *K. drynarioides* (Kodry), and *K. kauaiensis* (Kokau) and the sister clade *Gossipioides kirkii* (Gokir). Median values are displayed as a black triangle.

**
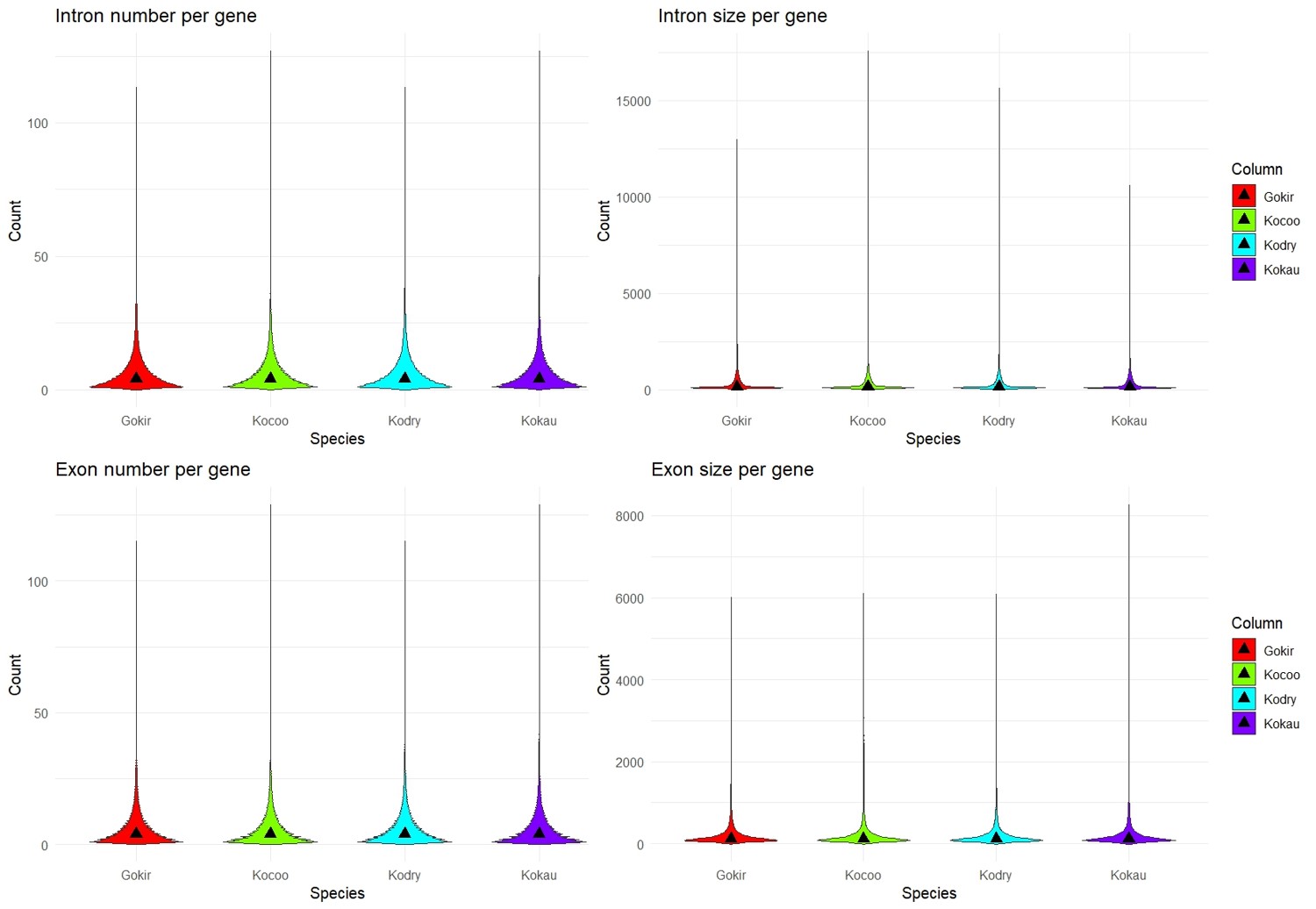
**
