# Supplementary material for: Genomic diversity and evolution of the Hawaiian Islands endemic Kokia (Malvaceae)
Source: G3 (Bethesda). 2024 Aug 6;14(10):jkae180. doi: 10.1093/g3journal/jkae180 (PMC11457090; doi:10.1093/g3journal/jkae180)
Supplement: jkae180_Supplementary_Data [file jkae180_supplementary_data.zip › Figure_S3_G3-2024-405044.docx]

**Figure S3:** Distribution of synonymous (d*S*) and non-synonymous (d*N*) substitution rates, as well as the ratio d*N*/d*S* between pairwise comparisons of the three *Kokia* genomes reported here and *Gossipioides kirkii*. The curve represents the frequency distribution of pairwise d*S* comparisons calculated for 17,224 single copy orthologs (identified by OrthoFinder v.2.5.4) with CODEML (PAML v.4.10.7) under the basic model (model = 0; NSsites = 0), after removing those with d*S* >1. Gokir/Gk: *Gossipioides kirkii*; Kocoo/Kc: *Kokia cookei*; Kodry/Kd: *K. drynarioides*; Kokau/Kk: *K. kauaiensis*.

**
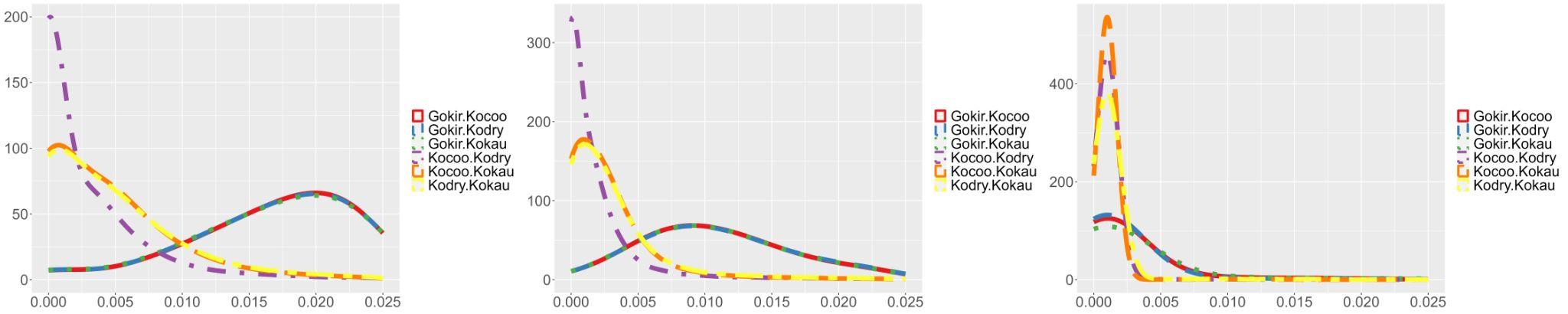
**
